# Differential impacts of TNFα inhibitors on the transcriptome of Th cells

**DOI:** 10.1186/s13075-021-02558-z

**Published:** 2021-07-23

**Authors:** Ching-Huang Ho, Andrea A. Silva, Beverly Tomita, Hui-Ying Weng, I-Cheng Ho

**Affiliations:** 1 Division of Rheumatology, Inflammation, and Immunity, Department of Medicine, Brigham and Women’s Hospital, 60 Fenwood Road, Boston, MA 02115 USA; 2 Harvard Medical School, 60 Fenwood Road, Boston, MA 02115 USA; 3 Biomedical Industry PhD Program, School of Life Sciences, National Yang-Ming University, Taipei, Taiwan

**Keywords:** TNFα inhibitors, Th cells, Cytokines, Type 1 interferon

## Abstract

**Background:**

Targeting TNFα is beneficial in many autoimmune and inflammatory diseases, including rheumatoid arthritis. However, the response to each of the existing TNFα inhibitors (TNFis) can be patient- and/or disease-dependent. In addition, TNFis can induce the production of type 1 interferons (IFNs), which contribute to their non-infection side effects, such as pustular psoriasis. Thus far, the molecular mechanisms mediating the drug-specific effects of TNFis and their induction of type 1 IFNs are not fully understood.

**Methods:**

Peripheral blood mononuclear cells (PBMCs) were collected from healthy donors and stimulated in vitro with anti-CD3 and anti-CD28 in the absence or presence of adalimumab, etanercept, or certolizumab. Th cells were isolated from the stimulated PBMCs, and their RNA was subjected to RNA-seq and quantitative polymerase chain reaction.

**Results:**

Adalimumab and etanercept, which contain Fc, but not certolizumab, which does not contain Fc, inhibited the expression of several effector cytokines by Th cells within anti-CD3/anti-CD28-stimulated PBMCs. Transcriptomic analyses further showed that adalimumab, but not certolizumab, reciprocally induced type 1 IFN signals and the expression of CD96 and SIRPG in Th cells. The unique effects of adalimumab were not due to preferential neutralization of soluble TNFα but instead were mediated by several distinct mechanisms independent or dependent of Fc-facilitated physical interaction between Th cells and CD14+ monocytes.

**Conclusions:**

TNFis can have drug-specific effects on the transcriptional profile of Th cells.

**Supplementary Information:**

The online version contains supplementary material available at 10.1186/s13075-021-02558-z.

## Background

TNFα inhibitors (TNFis) have emerged as one of the most effective classes of drugs for inflammatory arthritis, including rheumatoid arthritis, psoriatic arthritis, and ankylosing spondylitis. Thus far, there are five commonly used TNFis [1], including adalimumab (ada), golimumab (gol), etanercept (eta), certolizumab pegol (cert), and infliximab (inf). Ada and gol are human IgG1; eta contains recombinant human trimeric type 2 TNFα receptors fused to human IgG1 Fc; cert is pegylated Fab’ without Fc; inf is human-mouse chimeric IgG1. While there exist differences in affinity and binding valency to TNFα among TNFis, their efficacy and safety profile in rheumatoid arthritis are very comparable. However, some patients may respond to one TNFi but not the others; eta, unlike other TNFis, is not effective for Crohn’s disease or uveitis. The causes of these discrepant clinical observations are still unclear. In addition to higher risk of infection, post-hoc data has uncovered several unexpected side effects of TNFis, including anti-nuclear antibody (ANA), lupus-like diseases, demyelinating diseases, and pustular psoriasis. While there is no head-to-head comparison, cert may be less likely to induce ANA, lupus-like disease, and demyelinating diseases [2–5]. Furthermore, TNFis can induce type 1 IFN signals, which at least partly explain some of their non-infection side effects [6, 7]. How TNFis induce type 1 IFN signals is still not fully understood. The existing data suggests that neutralization of TNFα prevents the maturation of plasmacytoid dendritic cells (pDCs), which produce a high level of type 1 IFN but lose this ability upon maturation. TNFis therefore sustain the production of type 1 IFN by immature pDCs [6, 8]; however, additional mechanisms very likely exist. Elucidating the molecular mechanisms mediating the differences in bioactivity among TNFis and the induction of type 1 IFN signals will improve the efficacy and safety of treatments and bring us one step closer to personalized medicine in inflammatory arthritis.

TNFα is synthesized as a membrane-bound precursor of 233 amino acid residues (mTNFα), which is then cleaved by metalloproteases to become soluble TNFα (sTNFα) of 157 amino acid residues [9]. sTNFα in trimeric form can bind to type 1 and type 2 TNFα receptors (TNFαRs). TNFαR1 is constitutively expressed in many types of cells, activates NF-kB, AP-1, and caspases in TNFαR1-expressing cells, and is the main mediator of the pro-inflammatory effects of TNFα. The expression of TNFαR2 is limited to immune cells and its contribution to TNFα-induced inflammation is not fully clarified. Emerging data has convincingly demonstrated that mTNFα can also function as a receptor and upon engagement with TNFαR1 or TNFαR2 triggers signaling events in mTNFα-expressing cells, a process known as reverse signaling [10]. Reverse signaling through mTNFα has been shown to induce the expression of E-selectin in human Th cells [11], activate NF-kB driven-transcription in B lymphoma cells [12], enhance the production of TNFα in monocytes [13], and synergize with IL-2 to augment cytotoxicity of NK cells [14]. Reverse signaling through mTNFα can also be triggered by the TFNis [10], resulting in apoptosis of T cells [15, 16], degranulation of neutrophils [15, 16], and induction of TGF-β in macrophages [17]. Such reverse signaling events may contribute to the discordance in the therapeutic and/or side effects among the TNFis. Despite the observations, the functional consequence of mTNFα reverse signaling is still not fully understood.

Here we report that ada has effects on the transcriptome of primary human Th cells that are not seen with cert, including down-regulation of effector Th cytokines and induction of type 1 IFN signals. The unique effects of ada are not due to preferential neutralization of sTNFα and are mediated by at least three distinct mechanisms. Our data therefore suggest the presence of novel pathways for regulating the expression of Th cytokines and TNFi-induced production of type 1 IFN.

## Methods

### Human subjects

Healthy donor PBMCs were purified from leukoreduction collars obtained from the Crimson Biomaterials Collection Core Facility, which prospectively collects discarded clinical materials matching investigator-defined criteria against available information on clinical samples.

### Study approval

This study has been approved by Partners Human Research Committee (PHRC), Boston, MA. Informed consent was obtained from participants prior to inclusion to the studies.

### Purification and stimulation of PBMCs and Th cells

PBMCs were isolated from leukoreduction collars by Ficoll-Paque PLUS (17-1440-03, GE Healthcare, Pittsburgh, PA) density gradient centrifugation and cryopreserved prior to use. PBMCs were then thawed and plated in 24-well plates (2–2.5 millions/1 ml/well) pre-coated with anti-CD3 (2.5 μg/ml, HIT3a clone, Biolegend, San Diego, CA) in the presence of absence of soluble anti-CD28 (2 μg/ml, Cat. #302914, Biolegend) as well as adalimumab, etanercept, certolizumab pegol, and tocilizumab at indicated concentrations for 24 h before harvesting. In some experiments, Th (CD4+) cells were purified from resting PBMCs by using the human CD4+ T cell isolation kit (Cat. #130-045-101, Miltenyi Biotec, Bergisch, Gladbach, Germany) first before stimulation with anti-CD3 and anti-CD28. In the transwell experiments, Th cells were purified from 5 × 10^6^ of PBMCs and seeded on anti-CD3-coated inserts, whereas the remaining non-Th cells were plated in the bottom chambers of 24-well transwell plates (#140620, ThermoFisher, Waltham, MA). Exclusion of non-Th subsets from PBMCs was carried out with CD8 (#130-045-201, Miltenyi), CD19 (#130-050-301, Miltenyi), or CD14 (#130-050-201, Miltenyi) MicroBeads.

### Synovial fibroblasts

Human fibroblast like synoviocytes were a gift of Dr. Hung Nguyen and were cultivated in DMEM supplemented with 1% penicillin, 1% streptomycin, and 1% glutamine (Gibco) before stimulation with TNFα (10 ng/ml, PHC3015, Life Technology, Carlsbad, CA) for 24 h before harvest.

### RNA-seq and data analysis

RNA was prepared from sorted CD4+ Th by using Direct-zol RNA microprep kits (#R2063, Zymo, Irvine, CA), and the concentration and quality were checked by spectrophotometry. RNA samples were delivered to Admera Health (South Plainfield, NJ) or Broad institute (Cambridge, MA) for TruSeq RNA library preparation (Illumina, San Diego, CA) and bulk RNA sequencing. Results were returned in FASTQ format, and transcript-level of RNA-seq analysis was performed using HISAT2-StringTie-Ballgown workflow. Differentially expressed genes were identified with Qlucore Omics Explorer (Lund, Sweden).

### Quantitative RNA analysis

RNA isolation, reverse transcription, and quantitative PCR (qPCR) were performed as previously described [18]. The transcript levels thus detected were normalized against that of actin from the same sample. The sequences of the primers used in qPCR are listed in Supplemental Table 3.

### ELISA

Sandwich ELISA was performed using the following kits: human IL-17A ELISA set (#433914, Biolegend), human IL-17F ELISA set (#DY-1335B-05, R&D systems, Minneapolis, MN), and human IL-2 ELISA set (#431804, Biolegend). All ELISA experiments were performed according to manufacturer’s instructions.

### FACS

PBMCs were stained with anti-CD4 (#317450 and #300508, Biolegend), anti-CD8 (#344750 and #344714, Biolegend), anti-CD14 (#301815, Biolegend), anti-CD19 (#332224, Biolegend), anti-CD69 (#310904, Biolegend), anti-SIRPG (#336606, Biolegend), anti-CD96 (#338405, Biolegend), AF647- (#A20186, Invitrogen, Carlsbad, CA) conjugated adalimumab and control IgG1 (#403502, Biolegend). Stained cells were collected with FACSCanto or LSRFortessa (Becton Dickenson, Franklin Lakes, NJ), and the data was analyzed with FlowJo software (Becton Dickinson)

### Statistical analyses

Statistical analyses were carried out with one-way ANOVA followed by multiple comparisons (Figs. 1, 3, 4H and 6D, and Supplemental Figures 1 and 4), unpaired two-tailed Student’s t test (Figs. 2A and 5A), and paired two-tailed Student’s t test (Figs. 5C–E and 6B, C). The data shown in bar graphs is mean and SEM.

**Fig. 1:**
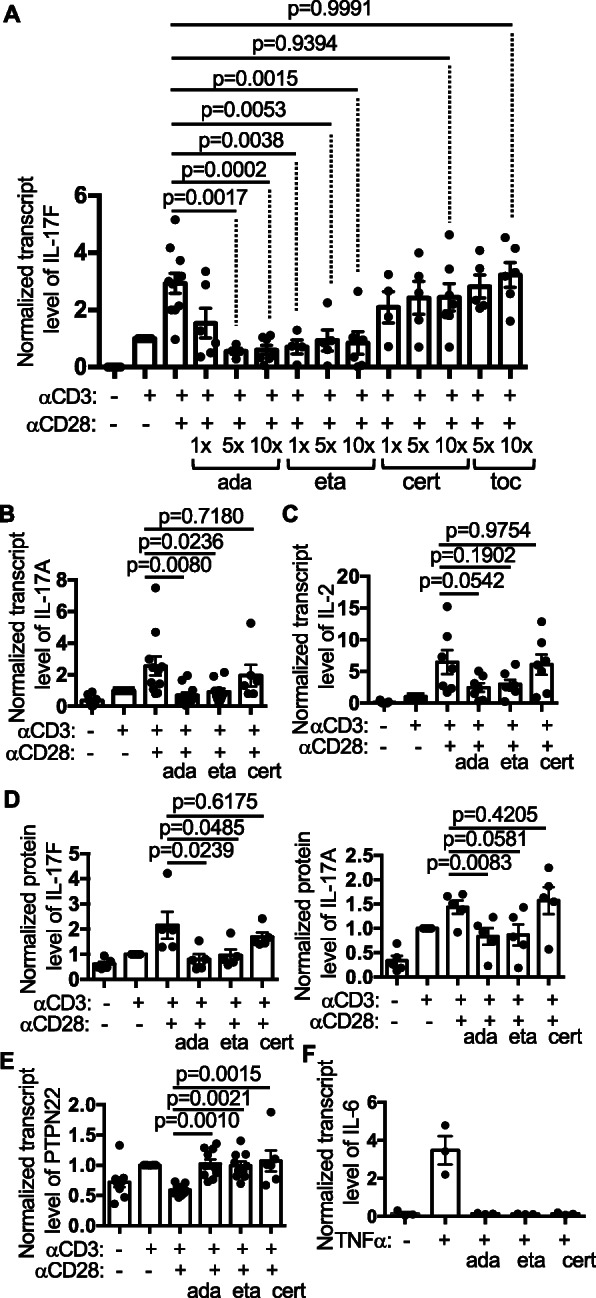
Differential impacts of TNFis on the expression of Th cytokines. **A**–**E** PBMCs from healthy donors were stimulated with anti-CD3 and anti-CD28 for 24 h in the absence or presence of indicated drugs at indicated concentration. In **A**, 1X equals 25 μg/ml for ada, 10 μg/ml for eta, 10 μg/ml for cert, and 20 μg/ml for toc. 5X was used for all drugs in **B**–**E**. The transcript levels of the indicated genes in the PBMCs were measured with qPCR (**A**–**C**, **E**), and the protein levels of IL17F and IL17A in the supernatant were quantified with ELISA (**D**). The levels of anti-CD3-stimulated samples were arbitrarily set as 1. In **D**, the level of 1 ranges from 26 to 137 pg/ml for IL-17F and 66-343 pg/ml for IL-17A. **F**. Human synovial fibroblasts were stimulated with TNFα (10 ng/ml) for 6 h in the presence or absence of the indicated TNFis at 5X concentration. The transcript level of IL-6 was measured with qPCR (N = 3)

**Fig. 2:**
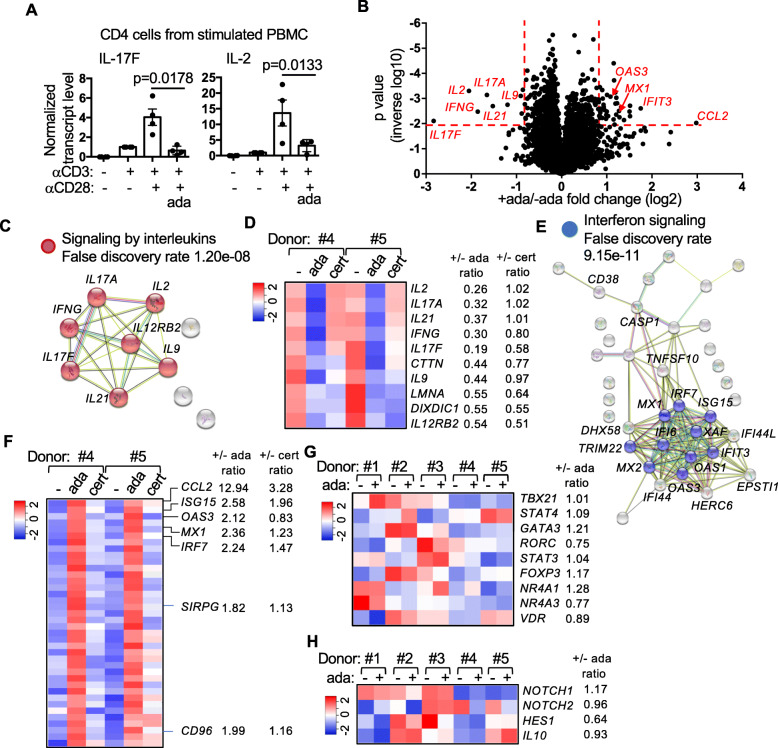
Differential impacts of TNFis on the transcriptome of Th cells. **A** Th cells were purified from PBMCs pre-stimulated in the presence or absence of 5X ada. The transcript levels of IL-17F and IL-2 were quantified with qPCR. The levels of anti-CD3-stimulated samples were arbitrarily set as 1. **B**–**G** Th cells were purified from PBMCs (5 donors) pre-stimulated with anti-CD3/anti-CD28 in the presence or absence of 5X ada (all 5 donors) or cert (donor #4 and #5) and subjected to RNA-seq. A volcano plot of genes whose expression was inhibited or induced by ada in all 5 donors are shown in **B**. The red dotted lines indicate p ≤ 0.015 and fold change ≥ 1.8 fold. The STRING reactomes of ada-suppressed and ada-induced genes are shown in **C** and **E**, respectively. The type 1 IFN-inducible genes identified by Interferome are indicated in **E**. The effects of ada and cert on the expression of the ada-regulated genes according to the RNA-seq data from donor #4 and #5 are compared in the heatmaps shown in **D** and **F**. The fold changes (+drug/-drug ratios) in the transcript levels are shown on the right of the heatmaps. Heatmaps displaying the effect of ada on the expression of the indicated transcription factors (**G**), Notch pathway, and IL-10 (**H**) from all 5 donors are also shown

**Fig. 3:**
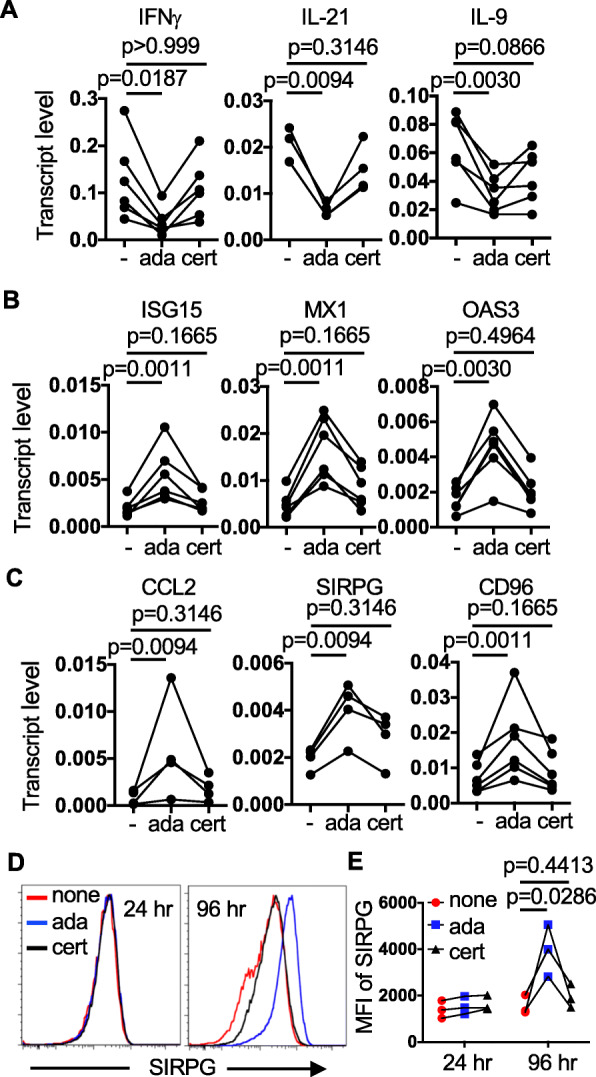
Confirmation of the differential impacts of TNFis on gene expression. **A**–**E** RNA was prepared from Th cells purified from PBMCs pre-stimulated with anti-CD3/anti-CD28 in the absence (-) or presence of indicated TNFis (5X). The transcript levels of Th cytokines (**A**), type 1 IFN-inducible genes (**B**), and CCL2, SIRPG, as well as CD96 (**C**) were quantified with qPCR. **D** & **E** A fraction of the stimulated PBMCs were subjected to FACS at indicated time points. The histograms of surface SIRPG levels among CD4+ Th cells are shown in **D**. The MFI of SIRPG from three independent experiments is shown in **E**

**Fig. 4:**
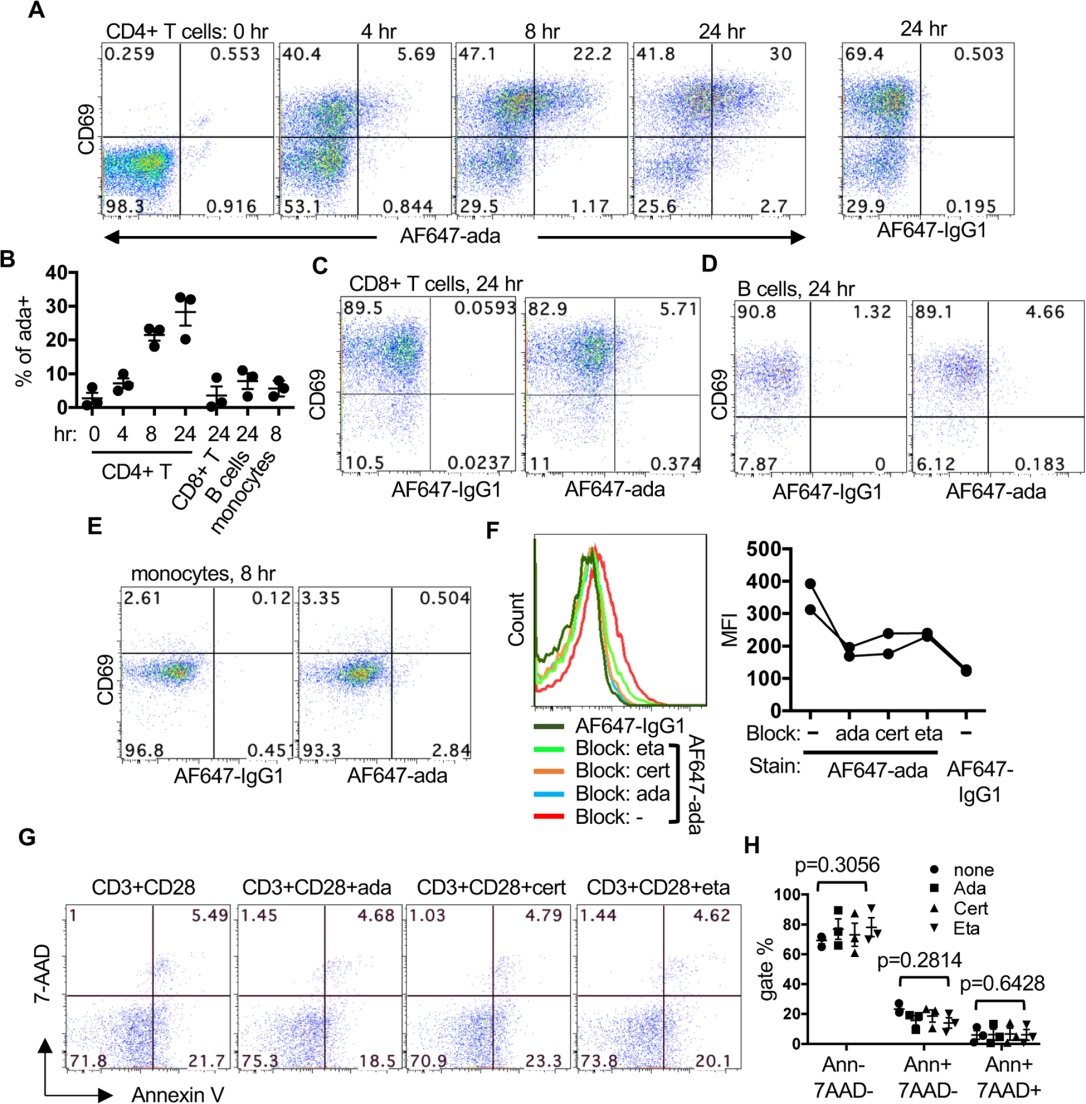
Comparable binding of TNFis to mTNFα. **A**–**F** PBMCs were stimulated with anti-CD3/anti-CD28 and stained with anti-CD69, AF647-ada or AF647-IgG1 at indicated time points. Representative FACS plots of CD4 + T cells are shown in **A**. Percentages of AF647-ada + Th cells from three experiments are shown in **B**. Representative FACS plots of CD8+ T cells, B cells, and monocytes are shown in **C**, **D**, and **E**. Unlabeled ada, eta, or cert was used to compete with AF647-ada staining in Th cells (**F**). Representative histograms from the 24-h time point are shown in the left panel and the MFI of AF647 from two experiments are shown in the right panel. **G** & **H** Apoptosis of Th cells from PBMCs pre-stimulated in the presence or absence of indicated TNFis was analyzed with FACS using AnnexinV and 7-AAD. Representative FACS plots (**G**) and percentages of indicated apoptotic populations (**H**) from three experiments are shown

**Fig. 5:**
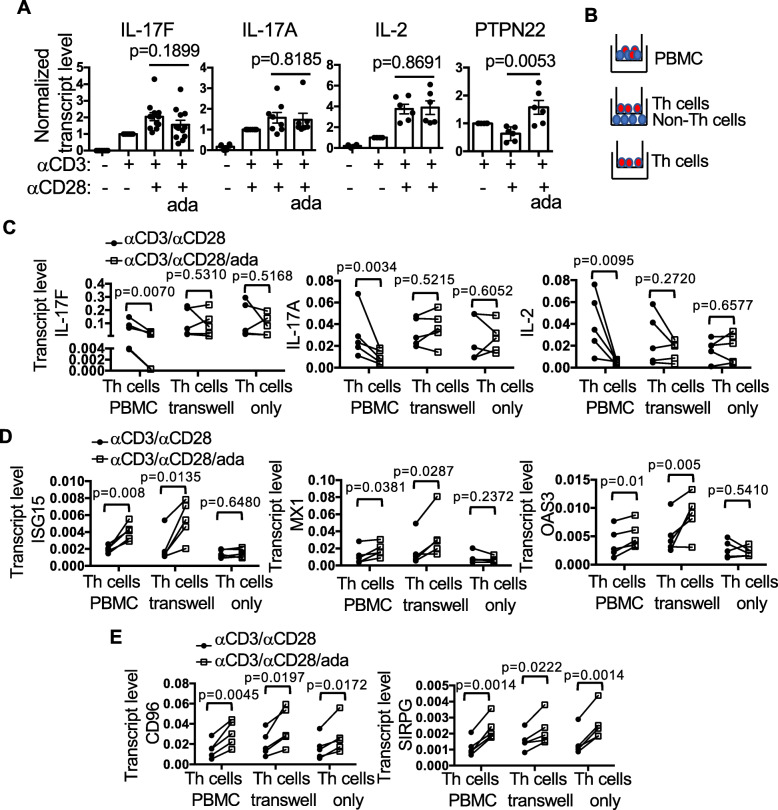
Non-Th cell-dependent and independent mechanisms of ada. **A** Th cells were first purified from resting PBMCs then stimulated in the presence of absence of ada (5X). The transcript levels of indicated genes were measured with qPCR. The levels of anti-CD3-stimulated samples were arbitrarily set as 1. **B**–**E** Th cells in the context of PBMCs, separated from non-Th cells in transwells, or without non-Th cells were stimulated in the absence or presence of ada (5X) for 24 h. A schematic diagram of the experimental design is illustrated in **B**. Th cells were then purified and their expression of cytokine genes (**C**), type 1 IFN-inducible genes (**D**), as well as CD96 and SIRPG (**E**) was examined with qPCR

**Fig. 6:**
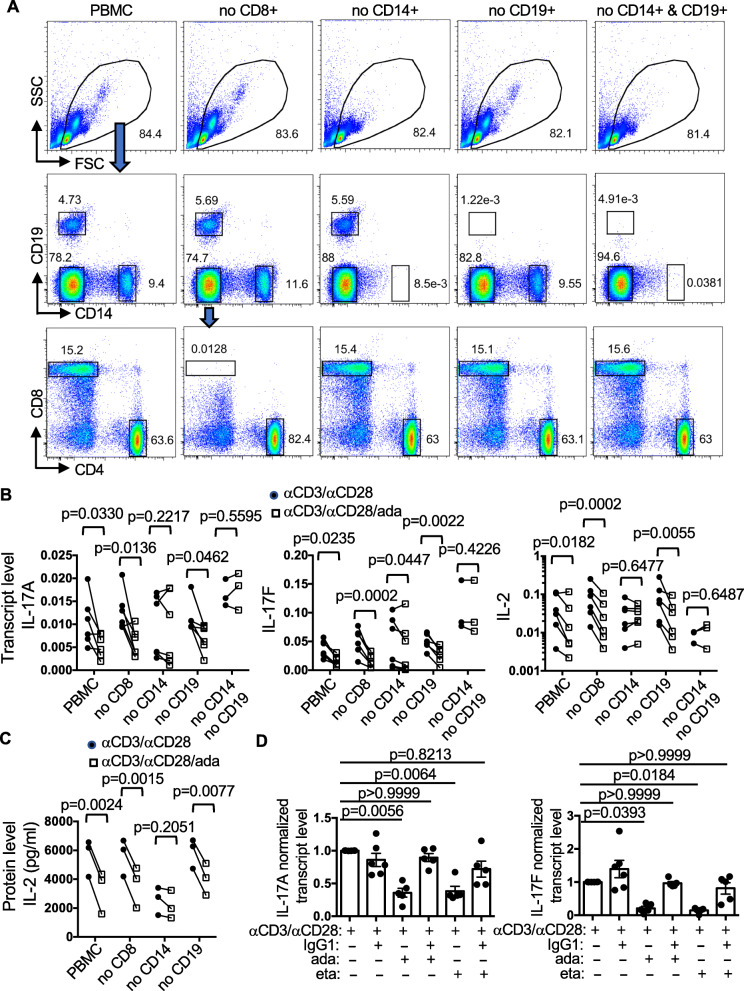
Dependence of CD14+ cells and Fc-mediated interaction for the effect of ada. **A**–**C** PBMCs were depleted of indicated populations through negative selection. The depletion was confirmed with FACS. Representative FACS plots are shown in **A**. The depleted PBMCs were stimulated with anti-CD3/anti-CD28 in the presence or absence of ada (5X). The transcript levels of indicated cytokines in the stimulated PBMCs were quantified with qPCR (**B**). Supernatant from three of the experiments was also subjected to IL-2 ELISA (**C**). **D** PBMCs were pre-treated with control human IgG1 (100 μg/ml) for 30 min, and then stimulated with anti-CD3/anti-CD28 in the presence or absence of ada (5X) or eta (5X) for 24 h. The transcript levels of indicated cytokines in PBMCs were measured with qPCR. The levels of samples stimulated in the absence of IgG1 or TNFi were arbitrarily set as 1

## Results

### Differential impacts of TNFis on the expression of Th cytokines

We have previously shown that both anti-CD28 and soluble TNFα inhibited the expression of PTPN22 [19], a gene that is associated with several autoimmune diseases [20], but only anti-CD28 promoted the production of IL-17A/F in anti-CD3 stimulated PBMCs. Interestingly, the expression of IL-17F in activated PBMCs was inhibited by ada or eta in a dose-dependent manner (Fig. 1A) but not by tocilizumab, a monoclonal human IgG1 against human IL-6R. Ada or eta inhibited the expression of IL-17F at a concentration close to their therapeutic concentration (3.5–50 μg/ml). Surprisingly, cert failed to do so even at a concentration as high as 100 μg/ml (Fig. 1A). Similar trends were observed when we examined the expression of IL-17A and IL-2 (Fig. 1B, C). The discordance between cert and the other two TNFis was also reflected in the protein levels of IL-17A and IL-17F in the supernatant of the stimulated PBMCs (Fig. 1D). By contrast, the expression of IL-10 was not affected by any of the three TNFis (Supplemental Figure 1).

One possible explanation for the discordance between cert and the other two TNFis is that cert is less potent in neutralizing endogenous sTNFα induced by anti-CD3/anti-CD28. This scenario is unlikely because cert not only restored the expression of PTPN22 in PBMCs (Fig. 1E) but also inhibited TNFα-induced expression of IL-6 by synovial fibroblasts as efficiently as ada or eta (Fig. 1F). In addition, exogenous IL-2, which is critical for the proliferation of T cells, did not rescue the expression of IL17A either (Supplemental Figure 2).

### Differential impacts of adalimumab and certolizumab on the transcriptome of Th cells

Th cells are the main expressors of IL-17A/F and IL-2. Indeed, when we isolated Th cells from the stimulated PBMCs, we again found that the transcript levels of IL-17A/F and IL-2 in the purified Th cells were markedly reduced when the PMBCs were stimulated in the presence of ada (Fig. 2A). To examine the impact of ada on the gene expression profile of Th cells, we prepared RNA from Th cells isolated from PBMCs (N = 5), which were pre-stimulated with anti-CD3/anti-CD28 in the presence or absence of ada for 24 h. The RNA was then subjected to RNA-seq.

We subsequently used a threshold of false discovery rate ≤ 0.2 and fold change ≥ 1.8 (+drug/−drug ratio ≤ 0.55 or ≥ 1.8) to identified differentially expressed genes. The volcano plot of p values and fold changes was shown in Fig. 2B. There were only 10 genes, whose expression was downregulated by ada in all five donors (Fig. 2C and Supplemental Table 1). Satisfyingly, *IL17A*, *IL17F*, and *IL2* were among the most down-regulated genes. There was also reduction in *IFNG*, *IL9*, *IL21*, and *IL12RB2*. *IFNG*, *IL9*, and *IL21* are the signature cytokine genes of Th1, Th9, and Tfh cells, suggesting that the effect of ada is not limited to Th17 cells. Expectedly, STRING v11 [21] identified *signaling by interleukins* as the most significant reactome among the 10 genes (Fig. 2C). PBMCs from two of the five donors (donor #4 and #5) were also stimulated in the presence of cert, enabling us to examine whether the ada-regulated genes were concordantly or discordantly regulated by cert (Fig. 2D). We found that the expression of the cytokine genes was not affected by cert based on the 1.8-fold threshold. By contrast, *IL12RB2* and *DIXDC1* were comparably downregulated by ada and cert.

Reversely, there were 38 genes, whose expression was concordantly upregulated by ada in all five donors (Fig. 2E, F, and Supplemental Table 2). STRING identified *Interferon Signaling* as the most significant reactome among the 38 genes (Fig. 2E), which includes *MX1*, *IRF7*, *ISG15*, and *OAS3*. Indeed, 18 of the 38 genes are type 1 interferon (IFN) inducible genes in human T cells according to Interferome v2.01 [22]. The ada-upregulated genes also include *CCL2*, *SIRPG*, and *CD96*. The latter two genes are preferentially expressed in T cells according to Human Protein Atlas and have been implicated in regulating the function of T cells [23–25]. Among the 38 genes, only *CCL2* and *ISG15* were also induced by cert in donor #4 and #5 based on the 1.8-fold threshold; however, the fold increases were much lower compared to those caused by ada (Fig. 2F).

One possible explanation for the unique effect of ada is that ada, but not cert, inhibits the activation of Th cells. However, ada had no consistent effect on the expression of several transcription factors that are known to be induced in activated T cells and/or critical for the expression of the Th cytokines, such as *NR4A1*, *TBX21*, *GATA3*, and *RORC* (Fig. 2G). Nor did ada have any impact on the expression of other T cell activation genes, such as *PDCD1*, *ICOS*, or *FASLG*. (Supplemental Figure 3). Ada has been shown to inhibit the activation of T cells through modulating the expression/signaling of *NOTCH1* [26]; however, we did not detect any change in the transcript level of *NOTCH1* and *NOTCH2* as well as their downstream gene *HES1* in our RNA-seq (Fig. 2H). The transcript levels of *NOTCH3*, *NOTCH4*, and *HES2* are too low (< 1) for meaningful comparison. In agreement with the data shown in Supplemental Figure 1, ada had no impact on the expression of *IL10* (Fig. 2H).

We subsequently confirmed the differential expression of the cytokine genes, including *IFNG*, *IL21*, and *IL9* (Fig. 3A), several type 1 IFN-inducible genes, such as *ISG15*, *MX1*, and *OAS3* (Fig. 3B), as well as *CCL2*, *SIRPG*, and *CD96* (Fig. 3C) with qPCR in Th cells obtained from stimulated PBMCs. SIRPG is a receptor-type transmembrane glycoprotein that is expressed almost exclusively in T cells. The unique effect of ada on the transcript level of *SIRPG* at 24 h was also reflected in the surface level of SIRPG in Th cells 96 h after stimulation (Fig. 3D, E). A similar effect was observed when we examined the surface level of CD96 (Supplemental Figure 4). While the transcript levels of *CCL2* and *ISG15* were increased by both ada and cert in our RNA-seq analysis, their expression was induced only by ada but not cert when quantified with qPCR.

### Comparable binding of TNFis to mTNFα

We then set to investigate the mechanism mediating the unique effect of ada. In addition to neutralizing sTNFα, TNFis can also bind to mTNFα, thereby triggering reverse signaling. It is possible that the discordant effect between ada and cert is due to differential binding to mTNFα. We therefore labeled ada with AF647 and used the labeled ada to stain PBMCs in the presence of FcR block. While very weak staining was detected in Th cells within resting PBMCs, approximately 20–30% of Th (CD4+) cells were stained positive by ada when PBMCs were stimulated with anti-CD3/anti-CD28 for 24 h (Fig. 4A, B and Supplemental Figure 5). Kinetically, ada + Th cells started to appear in Th cells 4 h after stimulation and their percentage gradually increased over the course of 24 h. The staining was almost detected exclusively in CD69+ population. By contrast, much weaker ada staining was detected in CD8+ T cells (Fig. 4B, C) and B cells (Fig. 4B, D). Monocytes (CD4med, CD14+ and/or CD16+) were still identifiable 8 h, but not 24 h, after stimulation and also had weak staining of ada (Fig. 4B, E, and Supplemental Figure 5). The cause for the weak ada staining in non-Th cells is still unknown and could be due to low expression of TNFα and/or rapid cleavage of mTNFα. The ada staining of Th cells was comparably competed away by unlabeled ada, eta, or cert at a molar concentration equivalent to that of AF647-labeled ada (Fig. 4F), suggesting that ada indeed binds to mTNFα and that the discordant effect between ada/eta and cert is not due to differential binding to mTNFα. Both sTNFα and mTNFα can trigger activation-induced cell death of T cells through forward and reverse signaling, respectively [27–29]; however, we found that the three TNFis had no differential effect on apoptosis of Th cells in our system (Fig. 4G, H). Thus, the unique effect of ada and eta cannot be explained by a difference in apoptosis.

### Non-Th cell-dependent and independent effects of adalimumab

We then examined whether ada acted directly on Th cells to inhibit their expression of cytokines. We purified Th cells first and then stimulated the Th cells with anti-CD3/anti-CD28 in the presence or absence of ada. Surprisingly, ada was ineffective in inhibiting the expression of IL-17A/F and IL-2 in the absence of non-Th cells even though it still enhanced the expression of PTPN22 (Fig. 5A). This observation suggests that the effect of ada on the expression Th cytokines requires non-Th cells. To further determine whether physical contact between Th and non-Th cells was necessary for the action of ada, we set up a transwell co-culture system, in which purified Th cells were stimulated in the top chambers, whereas autologous non-Th cells were cultured in the bottom chambers (Fig. 5B). We found that ada failed to inhibit the expression of IL-17A/F or IL-2 by Th cells when the Th cells were stimulated without physical contact with non-Th cells (Fig. 5C, Th cells transwell) or in the absence of non-Th cells (Fig. 5C, Th cells only). By contrast, the levels of the Th cytokines were reduced in Th cells purified from PBMCs that were stimulated in the presence of ada (Fig. 5C, Th cells PBMC).

Th cells typically do not express type 1 IFN. Expectedly, ada did not induce type 1 IFN signals in Th cells in the absence of non-Th cells (Fig. 5D, Th cells only). However, ada was able to augment the expression of ISG15, MX1, and OAS3 in Th cells from the transwell (Fig. 5D, Th cells transwell). This result indicates that the source of type 1 IFN is non-Th cells and that the ada-induced expression of type 1 IFN by non-Th cells does not require physical interaction between Th and non-Th cells. By contrast, ada effectively enhanced the expression of CD96 and SIRPG in Th cells from the transwell system and also in the absence of non-Th cells (Fig. 5E).

### Dependence of CD14+ monocyte and Fc-FcR interaction for the effects of ada on Th cytokines

Type 1 IFN has been shown to constrain Th17 cells by acting directly on Th cells to produce IL- IL-10 [30, 31] or acting on myeloid cells to express IL-27 [32], which then inhibits the differentiation of Th17 cells. Thus, ada could inhibit the expression of Th cytokines through promoting the expression of type 1 IFN by non-Th cells. This scenario is unlikely because the action of type 1 IFN should not depend on physical contact between Th and non-Th cells. In addition, neither ada nor cert affected the expression of IL-10 by Th cells (Fig. 2H and Supplemental Figure 1). To further elucidate the mechanism of action of ada, we set to identify the non-Th cells that are required for ada to inhibit the expression of Th cytokines. We separately deleted CD8+, CD19+ or CD14+ cells from PBMCs (Fig. 6A). The depleted PBMCs were then stimulated with anti-CD3/anti-CD28 in the absence or presence of ada. Depletion of CD14+ cells alone or together with CD19+ completely abrogated the effect of ada on the expression of IL-17A and IL-2 (Fig. 6B). Ada still subtly inhibited the expression of IL-17F in the absence of CD14+ cells but this residual effect was wiped out by additional depletion of CD19+ cells. By contrast, depletion of CD8+ cells or CD19+ cells alone had little or no effect at all. Supernatant from three of the experiments was also subjected to IL-2 ELISA and the results were consistent with that obtained with qPCR (Fig. 6C). These results indicate that CD14+ cells, but not CD8+ or CD19+ cells, are essential for mediating the inhibitory effect of ada on the expression of Th cytokines. Both ada and eta, but not cert, contain the Fc of human IgG1. It is possible that the unique effect of ada and eta, once bound to mTNFα on Th cells, requires the interaction of their Fc with FcR expressed by CD14+ monocytes and/or other non-Th cells. In agreement with this scenario, we found that pre-treating PBMCs with control human IgG1, which does not compete with ada or eta for binding to mTNFα but is expected to compete with the two Fc-containing TNFis for interaction with FcR, completely blocked the inhibitory effect of ada and eta on the expression of IL-17A and IL-17F (Fig. 6D).

The interaction between Fc of ada and FcR of non-Th cells may cause cross-linking of T cell-bound ada, thereby inhibiting the expression of Th cytokines. However, we found that cross-linking of T cell-bound ada with plate bound antibodies against the Fc of human IgG1 was unable to inhibit the expression of cytokine in purified Th cells (Supplemental Figure 6). Taken together, these results indicate that the unique effect of ada and eta depends on the interaction between their Fc and FcR on non-Th cells and that the contribution of such Fc-FcR interaction is more than causing cross-linking of Th cell-bound ada or eta.

## Discussion

We have uncovered intriguing differences in the biological effect between ada and cert. While both TNFis comparably neutralize sTNFα and induce the expression of PTPN22, ada has additional impacts on the transcriptome of Th cells through at least three distinct mechanisms. It inhibits the expression of Th cytokines, an effect depending on its Fc and physical contact between Th cells and CD14+ monocytes (A in Supplemental Figure 7), induces type 1 IFN signals in Th cells, an effect depending on soluble factors from non-Th cells (B in Supplemental Figure 7), and enhances the expression of CD96 and SIRPG, an effect independent of non-Th cells (C in Supplemental Figure 7).

The observation that ada inhibits the production of Th cytokines only in stimulated PBMCs but not purified Th cells is also consistent with published data [26, 33]; however, several discrepancies exist between our results and the published data. Werner et al. reported that ada and inf at 50 μg/ml, a dose comparable to that used in our study, inhibited the expression of IL-17 and IFNγ in PBMCs stimulated with anti-CD3 for 48 h [26]. Unfortunately, cert was not examined in Werner’s study. Regardless, their data suggest that TNFis attenuates the level of transmembrane Notch-1 and the expression of its downstream gene Hes-1 in T cells. Their data further suggest that the attenuated Notch-1 signaling by TNFis leads to the inhibition of Th cell activation and consequently the expression of Th cytokines. While this putative mechanism can explain the need for physical contact between Th and non-Th cells, it is in conflict with several publications showing a positive role of Notch signals in the activation of T cells [34–37]. In addition, our RNA-seq data shows that the expression of Notch1/2 and their downstream gene Hes-1 was not affected by ada. Povoleri et al. reported that ada at 1 μg/ml had no effect on the expression of IL-17 and IFNγ by purified CD4+ T cells when analyzed 3 days after stimulation [33], a finding consistent with our finding that ada did not affect the cytokine expression in Th cells in the absence of non-Th cells. However, they showed that ada inhibited the activation of purified CD4+ T cells and induced the expression of IL-10. These observations are different from our data showing that the expression of IL-10 and several activation-induced genes, such as NR4A1 and PDCD1, in Th cells from stimulated PBMCs was not affected by TNFis. The cause of this discrepancy is unclear and could be due to differences in the duration of stimulation, absence or presence of non-Th cells, and/or dose of ada.

We stimulated PBMCs with plate-bound anti-CD3 and soluble anti-CD28 to optimize the activation of Th cells. This setting may overtake physiological interaction between CD28 on Th cells and B7 on non-Th cells, thereby potentially making it difficult to detect the impact of non-Th cells on the differential effects of TNFis. However, the data shown in Fig. 6 clearly demonstrates a critical role of physical contact between Th cells and non-Th cells, particularly CD14+ cells, in mediating the inhibitory effect of ada on the production of Th cytokines. Thus, non-Th cells are not redundant but instead actively contribute to the differential effect of TNFis in our culture system. Despite our results, it remains unclear why the physical interaction between Th and CD14+ monocytes is required for the inhibitory effect of ada on the expression of Th cytokines. Our data strongly suggest that this interaction is mediated mainly by the Fc of Th cell-bound ada and FcR on monocytes. Such Fc-FcR interaction may induce the production of a soluble factor or the expression of a co-inhibitory surface molecule by monocytes. The transwell experiment shown in Fig. 5 argues against the former scenario. It is intriguing to notice that the expression of CD96 and SIRPG is enhanced by ada. Both CD96 and SIRPG are expressed preferentially, if not exclusively, in T and NK cells [23, 24]. CD96 can function as a co-suppressor and is potentially a target of immune checkpoint therapy [25]. SIRPG has been shown to interact with CD47 [38], which is ubiquitously expressed, but may have additional ligands. Its function is still unclear but several SNPs at the *SIRPG* locus are associated with higher risk of type 1 diabetes [39, 40]. While the induction of CD96 and SIRPG by ada is independent of non-Th cells, the expression of their counter-receptors, such as CD111, CD155, and CD47, on monocytes may still depend on the Fc-FcR interaction. It will be of great interest to determine whether the CD96 and/or SIRPG pathway contribute to the inhibitory effect of ada on the expression of Th cytokines.

Alternatively, the Fc-FcR interaction may trigger reverse signaling through ada-bound mTNFα in Th cells. mTNFα reverse signaling can also be triggered by TNFis, resulting in different functional outcomes [10]. For example, inf, gol, and ada induce apoptosis in T cells, and this effect requires three serine residues within the intracellular domain of mTNFα [15, 16], whereas cert induces a mTNFα-dependent non-apoptotic type of cell death [15]. The discrepancy in the functional consequence of mTNFα reverse signaling induced by different TNFis could be due to differences in the affinity and/or binding valency of various TNFis. If the unique effect of ada on Th cytokine expression is due to reverse signaling through mTNFα in Th cells, our data will not only uncover a potentially novel consequence of ada-induced mTNFα reverse signaling, i.e., inhibiting the expression of Th cytokines, but also suggest that the interaction between Fc of TNFis and FcR on non-Th cells can heavily influence the outcome of mTNFα reverse signaling. In agreement with this latter point, inf and ada, but not cert, have been shown to inhibit the proliferation of CD4+ T cells activated by allogeneic PBMCs and induce a distinct CD14+/HLA-DR+ macrophage population in a Fc-dependent manner [41]. It is still unclear whether the inhibitory effect of ada and eta on Th cytokine production can translate into in vivo situation, where endogenous IgG1 may dampen the effect of the TNFis. We do not think the scenario shown in Supplemental Figure 7 occurs in peripheral blood. Instead, we envision that the scenario is playing out in tissue, such as draining lymph nodes or inflamed synovium, where Th cells are activated and/or the concentration of TNFα is high enough to attract TNFis, thereby altering the local ratio between TNFis and endogenous IgG1. Unfortunately, there is still little information on the relative tissue distribution of TNFis and endogenous IgG1 in vivo.

Long-term treatment with TNFis is known to induce type 1 IFN signals, which contribute to the development of some of the non-infection side effects of TNFis, such as pustular psoriasis [6, 7]. Existing data suggest that TNFis lead to an arrest of pDCs at an immature TNFα-producing stage [6, 8]. Our data, however, suggest that short-term exposure of stimulated PBMCs to ada but not cert also induces the expression of type 1 IFN, probably by non-Th cells. We still do not have physical evidence for the presence of type 1 IFN. Nor do we know how ada uniquely induces the type 1 IFN signals. As the type 1 IFN signals were also detected in Th cells from the transwell, its induction is independent of Th/non-Th interaction. One plausible scenario is that ada and cert, while binding comparably to mTNFα, trigger a distinct reverse signaling cascade inside non-Th cells, resulting in the expression of type 1 IFN. This scenario is consistent with published data showing that various TNFis can act directly on myeloid cells with discordant outcomes [42–44]. For example, eta is less effective in inhibiting LPS-induced expression of IL-1β by human monocytes, whereas cert does not elicit the release of myeloperoxidase from human polymorphonucleocytes [42]. If ada indeed induces the expression of type 1 IFN, better understanding of the molecular mechanism mediating this effect of ada may lead to novel approaches of inhibiting TNFis-induced production of type 1 IFN and preventing some of the non-infection side effects of TNFis.

## Conclusions

Adalimumab compared to certolizumab has an unique impact on the gene expression of Th cells through at least three distinct mechanisms that are independent of neutralizing soluble TNFα.

## Supplementary Information

**Additional file 1:.** Table S1.

**Additional file 2:.** Table S2.

**Additional file 3:.** Table S3.

**Additional file 4:.** Figures S1-S7.

## Data Availability

The raw RNA-seq data will be available at NCBI database or upon request to the corresponding author.

## Abbreviations

ada: Adalimumab
ANA: Anti-nuclear antibody
cert: Certolizumab
eta: Etanercept
gol: Golimumab
IFN: Interferon
inf: Infliximab
mTNFα: Membrane TNFα
PBMCs: Peripheral blood mononuclear cells
pDC: Plasmacytoid dendritic cells
qPCR: Quantitative polymerase chain reaction
sTNFα: Soluble TNFα
TNFαR: TNFα receptor
TNFi: TNFα inhibitor
Toc: Tocilizumab
