# Supplementary material for: Differential impacts of TNFα inhibitors on the transcriptome of Th cells
Source: Arthritis Res Ther. 2021 Jul 23;23:199. doi: 10.1186/s13075-021-02558-z (PMC8299604; doi:10.1186/s13075-021-02558-z)
Supplement: Supplementary file 4 — Additional file 4:. Figures S1-S7. [file 13075_2021_2558_MOESM4_ESM.pdf]

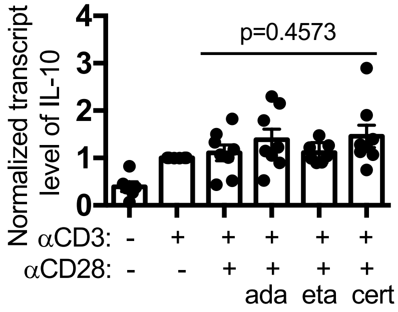

Supplemental Figure 1. Lack of effect of TNFis on the expression of IL-10 in stimulated PBMCs. The transcript level of IL-10 in PBMCs described in Figure 1B was quantified with qPCR.

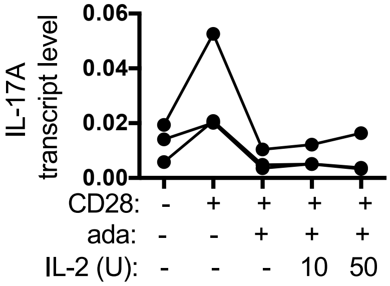

Supplemental Figure 2. Inability of exogenous IL-2 in counteracting the effect of ada. PBMCs were stimulated with anti-CD3 in the absence or presence of anti-CD28 (2 ug/ml), ada (5X), and human IL-2 for 24 hours. The transcript level of IL-17A was measured with qPCR (N=3).

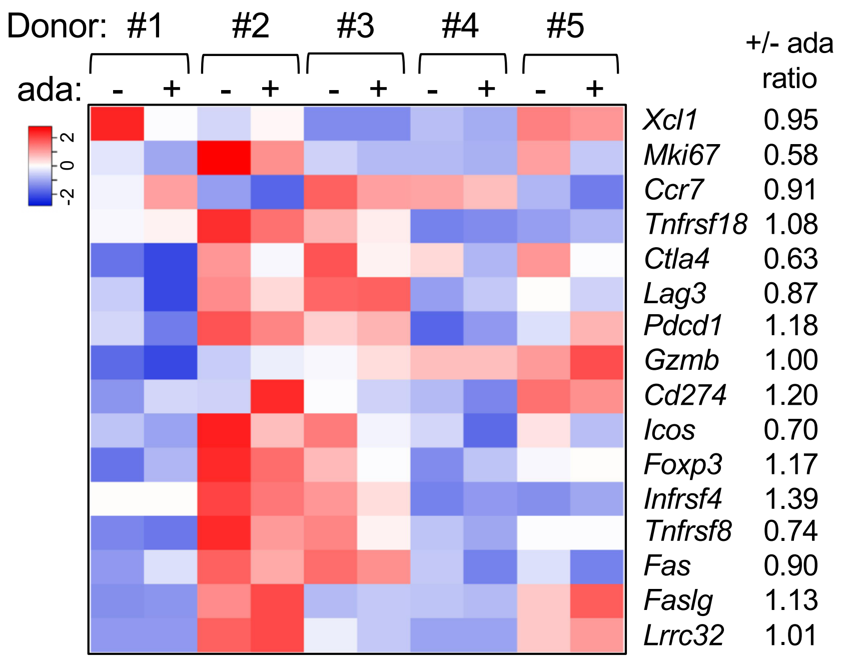

Supplemental Figure 3. Lack of effect of ada on the expression of activation genes in Th cells. The transcript reads of the indicated genes derived from the RNA-seq described in Figure 2B were displayed in a heatmap. The fold changes (+drug/-drug ratios) are shown on the right.

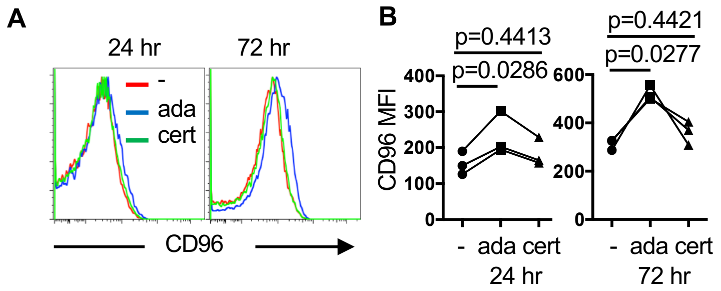

Supplemental Figure 4. Induction of CD96 by ada. PBMCs described in Figure 3D were stained with anti-CD96. Representative histograms of CD96 staining of Th cells at indicated time points are shown in **A**. The MFI of CD96 in Th cells from 3 experiments are shown in **B**.

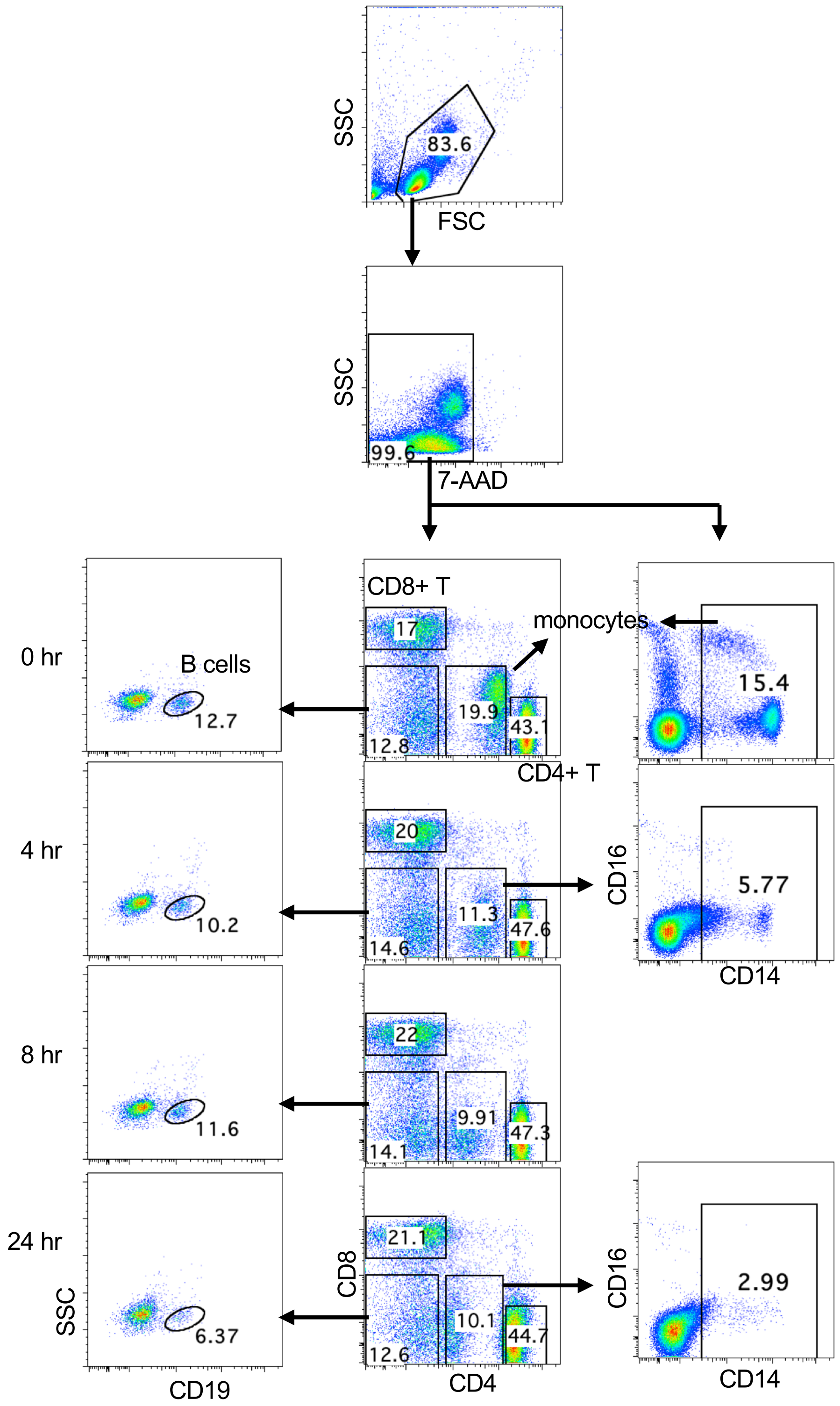

Supplemental Figure 5. Identification of various subsets of blood cells with FACS. PBMCs were stimulated with anti-CD3 and anti-CD28. The cells were stained with 7-AAD and indicated antibodies. Representative FACS plots and percentages of gated populations at indicated time points are shown.

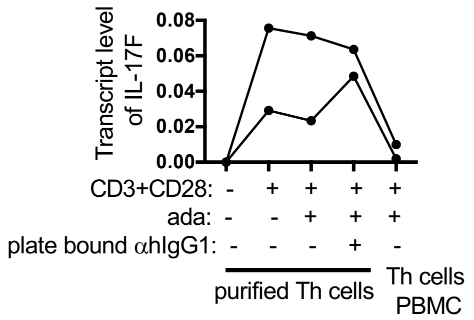

Supplemental Figure 6. Inability of ada cross-linking to suppress the expression of IL-17 in purified Th cells. Purified Th cells or autologous PBMCs were stimulated with anti-CD3/anti-CD28 in the absence (-) or presence of ada alone or along with plate-bound anti-Fc of human IgG1 for 24 hours. The transcript level of IL-17F in Th cells was quantified with qPCR (N=2).

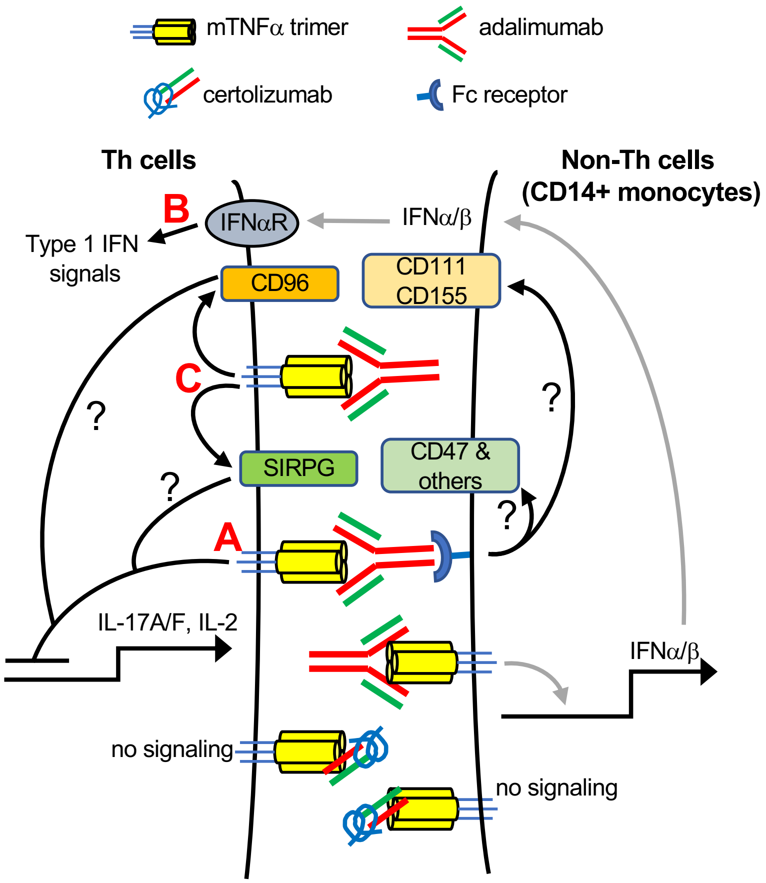

Supplemental Figure 7. A working model for the unique effects of adalimumab. The question marks indicate putative pathways not examined in this study. The gray arrows mark the pathways deduced from the data described in this study.
